# Intra- and inter-subtype HIV diversity between 1994 and 2018 in southern Uganda: a longitudinal population-based study

**DOI:** 10.1093/ve/veae065

**Published:** 2024-08-24

**Authors:** Seungwon Kim, Godfrey Kigozi, Michael A Martin, Ronald M Galiwango, Thomas C Quinn, Andrew D Redd, Robert Ssekubugu, David Bonsall, Deogratius Ssemwanga, Andrew Rambaut, Joshua T Herbeck, Steven J Reynolds, Brian Foley, Lucie Abeler-Dörner, Christophe Fraser, Oliver Ratmann, Joseph Kagaayi, Oliver Laeyendecker, Mary K Grabowski

**Affiliations:** Department of Pathology, Johns Hopkins School of Medicine, 600 N. Wolfe Street, Baltimore, MD 21205, United States; Research Department, Rakai Health Sciences Program, 4-6 Sanitary Lane, Old Bukoba Road, Kalisizo, Uganda; Department of Pathology, Johns Hopkins School of Medicine, 600 N. Wolfe Street, Baltimore, MD 21205, United States; Research Department, Rakai Health Sciences Program, 4-6 Sanitary Lane, Old Bukoba Road, Kalisizo, Uganda; Research Department, Rakai Health Sciences Program, 4-6 Sanitary Lane, Old Bukoba Road, Kalisizo, Uganda; Department of Medicine, Johns Hopkins School of Medicine, 600 N. Wolfe Street, Baltimore, MD 21205, United States; Division of Intramural Research, National Institute of Allergy and Infectious Diseases, National Institutes of Health, 5601 Fishers Lane, Bethesda, MD 20892, United States; Department of Medicine, Johns Hopkins School of Medicine, 600 N. Wolfe Street, Baltimore, MD 21205, United States; Division of Intramural Research, National Institute of Allergy and Infectious Diseases, National Institutes of Health, 5601 Fishers Lane, Bethesda, MD 20892, United States; Institute of Infectious Disease and Molecular Medicine, University of Cape Town, Anzio Road, Cape Town 7925, South Africa; Research Department, Rakai Health Sciences Program, 4-6 Sanitary Lane, Old Bukoba Road, Kalisizo, Uganda; Pandemic Sciences Institute, Nuffield Department of Medicine, University of Oxford, Old Road Campus, Oxford OX3 7DQ, United Kingdom; Wellcome Centre for Human Genetics, Nuffield Department of Medicine, University of Oxford, Roosevelt Drive, Oxford OX3 7BN, United Kingdom; Medical Research Council (MRC)/Uganda Virus Research Institute (UVRI) and London School of Hygiene and Tropical Medicine (LSHTM) Uganda Research Unit, Plot 51-59 Nakiwogo Road, Entebbe, Uganda; Uganda Virus Research Institute, Plot 51-59 Nakiwogo Road, Entebbe, Uganda; Institute of Evolutionary Biology, University of Edinburgh, Charlotte Auerbach Road, Edinburgh EH9 3FL, United Kingdom; Department of Global Health, University of Washington, 3980 15th Ave NE, Seattle, WA 98195, United States; Research Department, Rakai Health Sciences Program, 4-6 Sanitary Lane, Old Bukoba Road, Kalisizo, Uganda; Department of Medicine, Johns Hopkins School of Medicine, 600 N. Wolfe Street, Baltimore, MD 21205, United States; Division of Intramural Research, National Institute of Allergy and Infectious Diseases, National Institutes of Health, 5601 Fishers Lane, Bethesda, MD 20892, United States; Theoretical Biology and Biophysics, Los Alamos National Laboratory, P.O. Box 1663, Los Alamos, NM 87545, United States; Pandemic Sciences Institute, Nuffield Department of Medicine, University of Oxford, Old Road Campus, Oxford OX3 7DQ, United Kingdom; Pandemic Sciences Institute, Nuffield Department of Medicine, University of Oxford, Old Road Campus, Oxford OX3 7DQ, United Kingdom; Department of Mathematics, Imperial College London, 180 Queen’s Gate, London SW7 2AZ, United Kingdom; Research Department, Rakai Health Sciences Program, 4-6 Sanitary Lane, Old Bukoba Road, Kalisizo, Uganda; Department of Epidemiology, Makerere University School of Public Health, New Mulago Hill Road, Kampala, Uganda; Department of Medicine, Johns Hopkins School of Medicine, 600 N. Wolfe Street, Baltimore, MD 21205, United States; Division of Intramural Research, National Institute of Allergy and Infectious Diseases, National Institutes of Health, 5601 Fishers Lane, Bethesda, MD 20892, United States; Department of Epidemiology, Johns Hopkins Bloomberg School of Public Health, 615 N. Wolfe Street Baltimore, MD 21205, United States; Department of Pathology, Johns Hopkins School of Medicine, 600 N. Wolfe Street, Baltimore, MD 21205, United States; Research Department, Rakai Health Sciences Program, 4-6 Sanitary Lane, Old Bukoba Road, Kalisizo, Uganda; Department of Epidemiology, Johns Hopkins Bloomberg School of Public Health, 615 N. Wolfe Street Baltimore, MD 21205, United States

**Keywords:** HIV, genetic diversity, Uganda, subtype, recombinant

## Abstract

There is limited data on human immunodeficiency virus (HIV) evolutionary trends in African populations. We evaluated changes in HIV viral diversity and genetic divergence in southern Uganda over a 24-year period spanning the introduction and scale-up of HIV prevention and treatment programs using HIV sequence and survey data from the Rakai Community Cohort Study, an open longitudinal population-based HIV surveillance cohort. *Gag* (p24) and *env* (gp41) HIV data were generated from people living with HIV (PLHIV) in 31 inland semi-urban trading and agrarian communities (1994–2018) and four hyperendemic Lake Victoria fishing communities (2011–2018) under continuous surveillance. HIV subtype was assigned using the Recombination Identification Program with phylogenetic confirmation. Inter-subtype diversity was evaluated using the Shannon diversity index, and intra-subtype diversity with the nucleotide diversity and pairwise TN93 genetic distance. Genetic divergence was measured using root-to-tip distance and pairwise TN93 genetic distance analyses. Demographic history of HIV was inferred using a coalescent-based Bayesian Skygrid model. Evolutionary dynamics were assessed among demographic and behavioral population subgroups, including by migration status. 9931 HIV sequences were available from 4999 PLHIV, including 3060 and 1939 persons residing in inland and fishing communities, respectively. In inland communities, subtype A1 viruses proportionately increased from 14.3% in 1995 to 25.9% in 2017 (*P* < .001), while those of subtype D declined from 73.2% in 1995 to 28.2% in 2017 (*P* < .001). The proportion of viruses classified as recombinants significantly increased by nearly four-fold from 12.2% in 1995 to 44.8% in 2017. Inter-subtype HIV diversity has generally increased. While intra-subtype p24 genetic diversity and divergence leveled off after 2014, intra-subtype gp41 diversity, effective population size, and divergence increased through 2017. Intra- and inter-subtype viral diversity increased across all demographic and behavioral population subgroups, including among individuals with no recent migration history or extra-community sexual partners. This study provides insights into population-level HIV evolutionary dynamics following the scale-up of HIV prevention and treatment programs. Continued molecular surveillance may provide a better understanding of the dynamics driving population HIV evolution and yield important insights for epidemic control and vaccine development.

## Introduction

Human immunodeficiency virus type 1 (HIV-1) remains a significant public health threat. In 2022, an estimated 1.3 million new HIV cases and 630 000 AIDS-related deaths occurred globally, of which more than one-third were in eastern and southern Africa (UNAIDS 2023). Currently, there is no HIV vaccine to prevent HIV acquisition, partly due to extensive virus genetic diversity (Barouch and Korber 2010, Haynes et al. 2016, Ng’uni et al. 2020). Within the predominant HIV-1 group M viruses, there exist 17 distinct HIV-1 subtypes and sub-subtypes, and numerous inter- and intra-subtype recombinants forms (Taylor et al. 2008, Yamaguchi et al. 2020, Mendes Da Silva et al. 2021, LANL 2023). Similar to the global burden of cases and deaths, HIV genetic diversity is greatest in Africa (Vidal et al. 2000, Peeters et al. 2003, Lihana et al. 2012, Hemelaar et al. 2019). Thus, an effective preventive vaccine for African populations would most likely need to protect against a breadth of HIV subtypes and their derivatives (Burton et al. 2012, Burton and Hangartner 2016, Sadanand et al. 2016, Steichen et al. 2019).

Notwithstanding its significance for HIV vaccine and treatment development [e.g. broadly neutralizing antibodies (bNAbs)], data on accurate and up-to-date longitudinal trends in HIV genetic diversity are limited, particularly in eastern and southern Africa (Lihana et al. 2012, Hemelaar et al. 2019). Prior analyses of publicly available HIV sequence data, mostly conducted before widespread expansion of HIV treatment and prevention programs, showed increasing viral diversity in Africa as the epidemic grew (Peeters et al. 2003, Lihana et al. 2012, Hemelaar et al. 2019, 2020). However, spatially and temporally uneven sampling of HIV genomes may have biased these earlier assessments. Moreover, by the end of 2022, 83% of people living with HIV (PLHIV) in eastern and southern Africa were using antiretroviral therapy (ART), resulting in a decline of more than 57% in the number of new HIV infections since 2010 (UNAIDS 2023). It remains unclear to what extent such significant changes in the African HIV epidemic dynamics are associated with viral diversity within the region.

Understanding patterns in HIV evolution, particularly in response to roll-out of treatment and prevention programs, may provide insight into epidemic dynamics. For example, declining diversity might indicate that less common strains of virus have disappeared, or a concentration of transmission within population subgroups, resulting in smaller effective population size. In contrast, increasing diversity might highlight the growing role of virus introduction due to migration, having sex with non-local partners (Grabowski et al. 2014, Olawore et al. 2018), or signal diversifying selection.

Here, we used data from more than 30 continuously surveyed communities in southern Uganda over a nearly three-decade long period to evaluate trends in intra- and inter-subtype HIV viral diversity. Data were collected prior to and during the scale-up of HIV prevention and treatment programs through the Rakai Community Cohort Study (RCCS), an open population-based household census and HIV surveillance cohort. Following scale-up of ART and voluntary medical male circumcision programs beginning in 2004, HIV incidence rapidly declined in RCCS communities (Grabowski et al. 2017), consistent with regional trends (Joshi et al. 2021). RCCS communities have among the highest HIV case burdens worldwide (Chang et al. 2016), with extensive HIV subtype diversity, including co-circulation of HIV subtypes A1, C, and D and numerous recombinant forms (Arroyo et al. 2006, Conroy et al. 2010, Capoferri et al. 2020, Lamers et al. 2020). Building on previous work in the same population (Lamers et al. 2020), our study analyzed six additional years of data during a period of intense HIV intervention scale-up. While the previous study analyzed HIV sequence data in inland communities, we incorporated sequence and survey data from both inland and Lake Victoria fishing communities to evaluate trends in HIV genetic diversity using various metrics across a range of population subgroups. Our analyses provide insight into population-level HIV evolutionary dynamics in a high-burden region following HIV program scale-up and may inform the development and strategic deployment of HIV prevention and treatment interventions.

## Materials and methods

### Study design and population

The RCCS is conducted in inland agrarian and semi-urban trading communities (HIV prevalence 9%–26%) and hyperendemic Lake Victoria fishing communities (HIV prevalence 37%–43%) in southern Uganda (Chang et al. 2016). The RCCS has been ongoing since 1994 in inland communities and since 2011 in fishing communities. Longitudinal surveillance is based on a household census and individual survey conducted at ∼18 months intervals. The census enumerates all individuals in the household irrespective of age or presence in the community and documents residence status, including migrations into and out of the household. The survey follows the census and enrolls consenting adolescents and adults 15–49 years who are resident in the community. During the survey, demographic, sexual behavior (e.g. sex with partners outside community of residence), and HIV service utilization data (e.g. ART use and male circumcision status) are collected, and blood samples are obtained for HIV testing and molecular epidemiology (Wawer et al. 1998, Grabowski et al. 2017). RCCS participants are provided with HIV test results and offered free pre- and post-test counseling with referral to prevention and treatment services. All study participants provide written informed consent at baseline and follow-up visits. Here, we used HIV genomic sequence and survey data from PLHIV in 31 inland agrarian and semi-urban trading communities surveyed between November 1994 and May 2018 and four hyperendemic Lake Victoria fishing communities surveyed between November 2011 and August 2017.

### Sequencing

HIV sequence data have been generated using blood samples from RCCS participants living with HIV since the 1990s. Sequencing was attempted in some surveys for all participants with viremic HIV or not self-reporting ART use if viral load data were unavailable (Round 1: 1994–1995, Round 9: 2002–2003, and Rounds 13–18: 2008–2018) and only for selected participant visits for others. For blood samples collected prior to 2010, Sanger sequencing was used to generate consensus sequences for p24 in the *gag* gene (HXB2: 1249-1704) and gp41 in the *env* gene (HXB2: 7857-8260) (Yang et al. 2000). For blood samples collected from 2010 onward (beginning with survey round 14), near full-length genomes were generated using a next-generation sequencing approach in collaboration with the PANGEA-HIV consortium (Pillay et al. 2015, Abeler-Dörner et al. 2019) using the Gall protocol and the ve-SEQ HIV protocol (Gall et al. 2012, Bonsall et al. 2020). Resulting deep-sequencing reads were assembled using ‘shiver’ to generate HIV consensus sequences (Wymant et al. 2018). Near full-length HIV genomes collected between January 2010 and May 2018 were aligned using MAFFT (v7.52) and the HIV regions of p24 and gp41 were extracted from the aligned sequences to compare consensus HIV genome sequences between earlier and later surveys (Katoh and Standley 2013).

### Subtyping analyses

**Figure 1.**
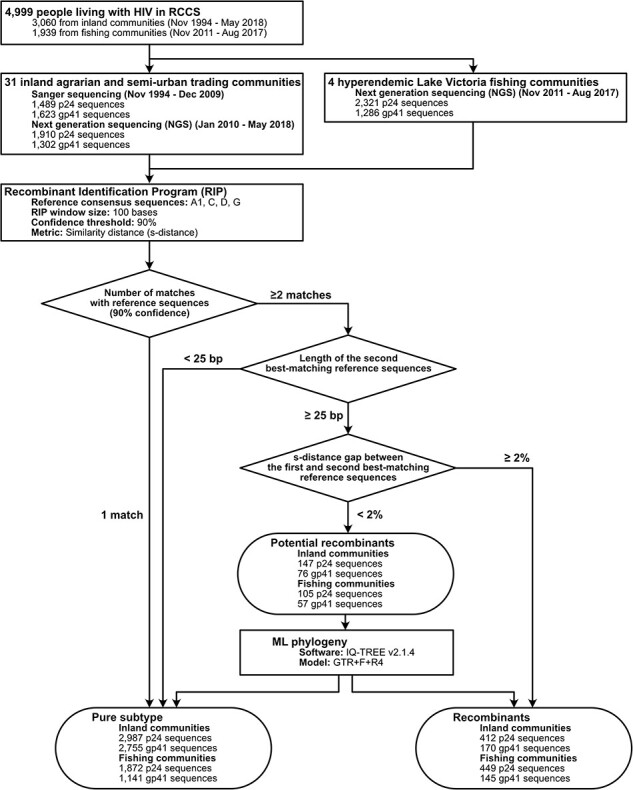
HIV genome sequence data collected from RCCS participants living with HIV in 31 inland agrarian and semi-urban trading communities and four hyperendemic Lake Victoria fishing communities and subtyping analyses using the RIP with phylogenetic confirmation.

The Recombinant Identification Program (RIP) (v3.0) was used to assign HIV subtypes to p24 and gp41 HIV genomes (Fig. 1) (Siepel et al. 1995). While COMET and REGA are two major HIV subtyping tools (Pineda-Peña et al. 2013, Struck et al. 2014), unlike RIP, they often failed to assign virus subtypes or identify recombinant viruses in p24 and gp41. In contrast, RIP analysis allowed for enhanced detection of recombinant viruses, which was critical for evaluating inter- and intra-subtype HIV genetic diversity trends in this study. Specifically, a RIP window of 100 bases and 90% confidence threshold was used to calculate similarity scores (*s*-distance: 1 − the Hamming distance between sequences within a RIP window) with the four most common circulating HIV-1 group M subtypes in Rakai (consensus reference sequences of A1, C, D, and G) (Supplementary Table S1). HIV sequences were assigned as a pure subtype if they had a single best match with one of the reference sequences with 90% confidence. If there were more than two matches with the reference sequences with 90% confidence in any region, the length of the second best-matching reference sequence was measured, and if it was shorter than 25 bases, viruses were classified as pure subtypes. Otherwise, the *s*-distance gap between the first and second best-matching reference sequences in the potential recombinant region was used to further classify them; if the gap was greater than 2%, sequences were subtyped as recombinants; otherwise, they were classified as potential recombinants. Next, pure, definitive, and potential recombinant sequences were aligned. Maximum Likelihood (ML) phylogenetic trees were generated from these alignments in IQ-TREE (v2.1.4) (Minh et al. 2020), and using resulting trees, recombinant sequences were classified as either pure subtype or recombinant viruses. We further analyzed HIV subtypes among participants who had available sequence data from both genes in the same surveys. HIV subtypes from these two gene regions were combined if the sequence data were obtained from the same individuals in the same survey round. For example, a participant with subtype A1 virus in both genes would be considered as having a subtype A1 infection, whereas a participant with subtype A1 in one gene and subtype D in the other would be considered an A1/D recombinant infection. The results from the RIP analysis with phylogenetic confirmation were compared to HIV subtype assignment based on COMET and REGA subtyping tools, both with a 70% of bootstrap support threshold (Supplementary Tables S2 and S3) (Hillis and Bull 1993, Pineda-Peña et al. 2013). The Cochran–Armitage test for trend in proportions was used to assess the statistical significance of longitudinal trends in HIV subtype distributions over calendar time using ‘stats’ package (v4.3.2) (part of base R) in R (v4.3.1) (R Core Team 2021).

### Diversity measures

#### Shannon diversity index

We next used the Shannon diversity index (SDI) to evaluate longitudinal trends in HIV inter-subtype diversity in inland and fishing communities. SDI quantifies biodiversity and species richness within a population (Shannon 1948). SDI was calculated as follows: $H^{\prime}= - \mathop \sum \limits_{i = 1}^S \left( {{p_i}\ln {p_i}} \right)$, where *S* is the total number of HIV subtypes in one survey round and ${p_i}$ is the proportion of subtype $i$ sequences relative to the total number of sequences in one survey round. A high $H^{\prime}$ value indicates greater inter-subtype diversity and more even distribution of sequences among subtypes, whereas a low $H^{\prime}$ implies lower intra-subtype diversity and the dominance of particular subtypes. For these analyses, inter-subtype recombinants of the same subtypes were classified as a single subtype irrespective of breakpoint.

We measured SDI in p24 and gp41 within inland and fishing communities overall and then within various population subgroups, including by sex, age group, occupation, and HIV-related risk behavior (numbers of past year sexual partners and sexual partners outside the community). We also assessed SDI trends among incident cases (i.e. participants testing positive for the first time with an HIV seronegative test result at the prior visit) and among persons with a recent migration history (∼1.5 years) into study communities. Occupations were grouped differently for inland (agricultural, trading/trucking, and other) and fishing communities (fisherfolk, bar/restaurant workers, and other) to reflect the distinct occupational structures between these two community types (Chang et al. 2016).

Temporal trends in SDI were evaluated using linear regression. We randomly selected 65% of HIV sequences from each survey and measured SDI using these resampled datasets. Then, we conducted regression analyses with SDI as the response variable and survey round as the predictor. We obtained ML regression coefficients from the resampled datasets, repeated this process 10 000 times, and then calculated the medians and 95% bootstrap confidence intervals (CIs) of the regression coefficients. SDI values were calculated using ‘poppr’ package (v2.9.4) and 95% bootstrap CIs were generated using ‘boot’ package (v1.3.28.1) (part of base R) in R (Kamvar et al. 2014).

#### Nucleotide diversity

We also estimated nucleotide diversity ($\pi $) and pairwise TN93 genetic distance in p24 and gp41 to measure longitudinal trends in inter- and intra-subtype genetic diversity. The nucleotide diversity index is a measure of genetic variation that quantifies the average number of nucleotide differences per site between all possible pairs of sequences in a population. $\pi $ was calculated as follows: $\hat \pi = \frac{n}{{n - 1}}\mathop \sum \limits_{ij} {x_i}{x_j}{\pi _{ij}}$, where *n* is the number of sequences, ${x_i}$ and ${x_j}$ are the frequencies of the *i*th and *j*th sequences, and ${\pi _{ij}}$ is the number of nucleotide differences per site between *i*th and *j*th sequences. First, the overall nucleotide diversity statistics, regardless of subtype, were calculated to evaluate the degree of genetic variation of HIV within the entire population in each survey round. Then, subtype-specific estimates within HIV subtypes A1 and D were measured to evaluate temporal trends in the genetic diversity of two most common HIV subtypes in our data set. Similar to SDI, we calculated HIV nucleotide diversity in the entire population and within various population subgroups. The medians and 95% bootstrap CIs of temporal trends in nucleotide diversity were measured using the same procedure as for SDI. Lastly, we conducted sensitivity analyses to evaluate the impact of a small sampling fraction in later surveys on nucleotide diversity. Assuming that ART initiation is independent of viral subtype, we randomly selected one-third of the sequences available in each survey, calculated nucleotide diversity, and repeated this process 1000 times. The medians and 95% bootstrap CIs of nucleotide diversity distribution obtained from these iterations were then compared with the nucleotide diversity estimates and 95% bootstrap CIs from the full sequence sets. Nucleotide diversity measures were calculated using ‘pegas’ package (v1.2) (Paradis 2010).

#### Genetic divergence

We further calculated root-to-tip distances and pairwise TN93 genetic distances to evaluate longitudinal trends of population-level HIV genetic divergence in p24 and gp41. First, subtype-specific (A1, C, and D) p24 and gp41 ML phylogenetic trees were reconstructed in IQ-TREE using the General Time Reversible model with empirical base frequencies and free rate model with four categories (GTR + F + R4) (Yang 1995). ML phylogenetic trees were rooted using sequences from other subtypes as the outgroup. Patristic distances from the root to the tips of phylogenetic trees (root-to-tip distances) were measured and plotted against the sampling times of sequences. The evolutionary rates and time to the most recent common ancestor were calculated using linear regression. Root-to-tip distances were calculated using ‘adephylo’ package (v1.1.16) (Jombart et al. 2010).

Additionally, we measured pairwise TN93 genetic distances of HIV subtypes A1, C, and D between the HIV consensus sequences of Los Alamos National Laboratory (LANL) HIV Database subtype references and HIV sequence data collected in Rakai to evaluate levels of HIV genetic divergence from the population consensus of corresponding HIV subtypes in each survey (Tamura and Nei 1993). LANL reference sequences of subtypes A1, C, and D (Supplementary Table S1) were retrieved, and the consensus sequences were generated using the Consensus Maker Tools (https://www.hiv.lanl.gov/content/sequence/CONSENSUS/consensus.html). Pairwise TN93 genetic distances were calculated using ‘ape’ package (v5.7.1) in R (Paradis et al. 2019).

#### Demographic history of HIV

We used a Bayesian Skygrid coalescent model to reconstruct the demographic history and epidemic growth of HIV using BEAST (v1.10.4) (Drummond et al. 2005, Lemey et al. 2009, Hill and Baele 2019). The posterior estimates of the effective population size were measured in unit of ${N_e}\tau $, where ${N_e}$ represents the effective population size and $\tau $ denotes the generation length in years. We randomly selected 40 sequences in p24 and gp41 subtypes A1 and D from each survey, irrespective of community type (*n* = 320). We used the HKY85+Γ nucleotide substitution model and an uncorrelated relaxed molecular clock with a lognormal distribution. Three independent analyses were conducted, each running for 300 million generations with sampling every 30 000 states. The resulting log files were combined after discarding the first 10% of states as burn-in using LogCombiner (v1.10.4). Convergence of parameters was assessed in Tracer (v1.6) (Rambaut et al. 2013), and effective sample sizes for all posterior estimates were greater than 200, indicating sufficient mixing of the Markov Chain Monte Carlo chains.

### HIV evolution and selective pressures

Lastly, we assessed if there was evidence of selective virus evolution. First, we calculated Tajima’s *D* statistic in each survey to quantify deviations from the expected genetic diversity under neutral evolution, where most of the genetic variation arises from neutral mutations. Tajima’s *D* is calculated as the normalized difference between the nucleotide genetic diversity and the expected number of segregating sites (Tajima 1989). A substantial deviation of Tajima’s *D* from 0 indicates the potential effects of natural selection, changes in population size, or other non-neutral processes. To prevent overestimation of segregating sites, p24 and gp41 sequences with gaps >10 bases were excluded for the calculation of Tajima’s *D* statistic. The *P*-values of Tajima’s *D* statistic were calculated using a beta distribution with a mean of zero and a variance of one, a conservative test as demonstrated by a previous simulation study (Fu and Li 1993). Tajima’s *D* statistic was calculated using ‘pegas’ package in R.

We further calculated the d*N*/d*S* ratio ($\omega $) to evaluate a population-level gene-wide selective pressure on HIV subtypes A1 and D p24 and gp41 (Yang and Bielawski 2000). This metric measures the ratio of nonsynonymous substitutions to synonymous substitutions in a gene to assess evolutionary forces acting on the gene within a population. A value of $\omega < 1$ suggests purifying or negative selection, indicating that nonsynonymous changes are being removed or are not favored within a population. A value of $\omega = 1$ implies neutral evolution, whereas $\omega > 1$ indicates positive or diversifying selection, suggesting that nonsynonymous changes are favored and occur more frequently than synonymous changes. To estimate $\omega $, we first reconstructed ML phylogenetic trees in IQ-TREE (v2.1.4) and from these trees, we calculated $\omega $ in codeml in the PAML package (v4.9 j) (Yang 2007). For the d*N*/d*S* ratio analyses, we randomly selected 50 sequences from each survey round (*n* = 400 in inland communities and *n* = 176 in fishing communities) to maintain computational efficiency. The mutation-selection model with observed codon frequencies was used to account for the codon composition and mutational bias (Yang and Nielsen 2008). Sensitivity analyses with five different random sequence sets were performed to assess the impact of downsampling on the estimated parameter values.

## Results

### Population characteristics

Between 1994 and 2018, 46 565 individuals participated in the RCCS of whom 37 606 lived in inland agrarian and semi-urban trading communities and 8959 in hyperendemic Lake Victoria fishing communities. Of these participants, 5352 (14.2%) in inland communities and 3402 (38.0%) in fishing communities were living with HIV. Among participants living with HIV, 3060 (57.2%) in inland communities and 1939 (57.0%) in fishing communities had HIV sequence data available in either p24 or gp41 genes from at least one survey visit. Of these participants with HIV sequence data available, 2111 (69.0%) in inland communities and 1121 (57.8%) in fishing communities had both p24 and gp41 sequence data. Individuals with HIV sequence data were less likely to be using ART, be male, and younger than individuals without HIV sequence data (Table 1; Supplementary Tables S4 and S5). Despite more participants living with HIV in later survey periods, HIV sequence data were less commonly available because of the scale-up of ART and resulting increases in HIV viral load suppression over calendar time.

**Table 1.** Demographic and HIV-related risk behavioral characteristics of RCCS participants living with HIV, with and without HIV sequence data in 31 inland agrarian and semi-urban trading communities between 1994 and 2018.

	R1 (November 1994–August 1995)			R13 (June 2008–December 2009)			R18 (October 2016–May 2018)		
Inland communities	PLHIV	PLHIV with sequence	PLHIV without sequence	PLHIV	PLHIV with sequence	PLHIV without sequence	PLHIV	PLHIV with sequence	PLHIV without sequence
*N*	1162	604	558	1218	703	515	1648	269	1379
Age (years)									
Median age (IQR)	29 (24, 35)	30 (24, 35)	28 (24, 35)	33 (28, 39)	31 (26, 37)	35 (31, 41)	36 (30, 42)	30 (25, 35)	37 (31, 42)
15–24	315 (27.1%)	154 (25.5%)	161 (28.9%)	170 (14%)	130 (18.5%)	40 (7.8%)	173 (10.5%)	66 (24.5%)	107 (7.8%)
25–34	531 (45.7%)	279 (46.2%)	252 (45.2%)	542 (44.5%)	346 (49.2%)	196 (38.1%)	559 (33.9%)	130 (48.3%)	429 (31.1%)
35+	316 (27.2%)	171 (28.3%)	145 (26%)	506 (41.5%)	227 (32.3%)	279 (54.2%)	916 (55.6%)	73 (27.1%)	843 (61.1%)
Sex									
Male	440 (37.9%)	251 (41.6%)	189 (33.9%)	425 (34.9%)	263 (37.4%)	162 (31.5%)	507 (30.8%)	116 (43.1%)	391 (28.4%)
Female	722 (62.1%)	353 (58.4%)	369 (66.1%)	793 (65.1%)	440 (62.6%)	353 (68.5%)	1141 (69.2%)	153 (56.9%)	988 (71.6%)
Occupation									
Agriculture	654 (56.3%)	331 (54.8%)	323 (57.9%)	637 (52.3%)	359 (51.1%)	278 (54%)	870 (52.8%)	115 (42.8%)	755 (54.7%)
Trade/truck	150 (12.9%)	90 (14.9%)	60 (10.8%)	168 (13.8%)	94 (13.4%)	74 (14.4%)	216 (13.1%)	52 (19.3%)	164 (11.9%)
Restaurant/bar	32 (2.8%)	18 (3%)	14 (2.5%)	58 (4.8%)	32 (4.6%)	26 (5%)	103 (6.2%)	10 (3.7%)	93 (6.7%)
Other	326 (28.1%)	165 (27.3%)	161 (28.9%)	355 (29.1%)	218 (31%)	137 (26.6%)	459 (27.9%)	92 (34.2%)	367 (26.6%)
Number of sexual partners in the past year									
0–1	959 (82.5%)	488 (80.8%)	471 (84.4%)	794 (65.2%)	478 (68%)	316 (61.4%)	1020 (61.9%)	149 (55.4%)	871 (63.2%)
2+	197 (17%)	114 (18.9%)	83 (14.9%)	239 (19.6%)	149 (21.2%)	90 (17.5%)	346 (21%)	82 (30.5%)	264 (19.1%)
No response	6 (0.5%)	2 (0.3%)	4 (0.7%)	185 (15.2%)	76 (10.8%)	109 (21.2%)	282 (17.1%)	38 (14.1%)	244 (17.7%)
Any sexual partner outside the community in the past year									
Yes	193 (16.6%)	101 (16.7%)	92 (16.5%)	333 (27.3%)	202 (28.7%)	131 (25.4%)	482 (29.2%)	101 (37.5%)	381 (27.6%)
No	968 (83.3%)	502 (83.1%)	466 (83.5%)	700 (57.5%)	425 (60.5%)	275 (53.4%)	884 (53.6%)	130 (48.3%)	754 (54.7%)
No response	1 (0.1%)	1 (0.2%)	0 (0%)	185 (15.2%)	76 (10.8%)	109 (21.2%)	282 (17.1%)	38 (14.1%)	244 (17.7%)
Recent history of migration									
Yes	0 (0%)	0 (0%)	0 (0%)	171 (14%)	113 (16.1%)	58 (11.3%)	405 (24.6%)	124 (46.1%)	281 (20.4%)
No	1115 (96%)	578 (95.7%)	537 (96.2%)	1045 (85.8%)	588 (83.6%)	457 (88.7%)	1243 (75.4%)	145 (53.9%)	1098 (79.6%)
Nonresident	47 (4%)	26 (4.3%)	21 (3.8%)	2 (0.2%)	2 (0.3%)	0 (0%)	0 (0%)	0 (0%)	0 (0%)
Self-reported ART use									
Yes	0 (0%)	0 (0%)	0 (0%)	268 (22%)	9 (1.3%)	259 (50.3%)	1277 (77.5%)	64 (23.8%)	1213 (88%)
No	0 (0%)	0 (0%)	0 (0%)	731 (60%)	524 (74.5%)	207 (40.2%)	332 (20.1%)	186 (69.1%)	146 (10.6%)
No response	0 (0%)	0 (0%)	0 (0%)	219 (18%)	170 (24.2%)	49 (9.5%)	39 (2.4%)	19 (7.1%)	20 (1.5%)
Not available	1162 (100%)	604 (100%)	558 (100%)	0 (0%)	0 (0%)	0 (0%)	0 (0%)	0 (0%)	0 (0%)
Viral load									
≥1000	462 (39.8%)	277 (45.9%)	185 (33.2%)	690 (56.7%)	573 (81.5%)	117 (22.7%)	312 (18.9%)	269 (100%)	43 (3.1%)
50 ≤ VL <1000	24 (2.1%)	11 (1.8%)	13 (2.3%)	117 (9.6%)	71 (10.1%)	46 (8.9%)	56 (3.4%)	0 (0%)	56 (4.1%)
BD	21 (1.8%)	2 (0.3%)	19 (3.4%)	355 (29.1%)	29 (4.1%)	326 (63.3%)	1264 (76.7%)	0 (0%)	1264 (91.7%)
No data	655 (56.4%)	314 (52%)	341 (61.1%)	56 (4.6%)	30 (4.3%)	26 (5%)	16 (1%)	0 (0%)	16 (1.2%)

### Subtype distribution

**Figure 2.**
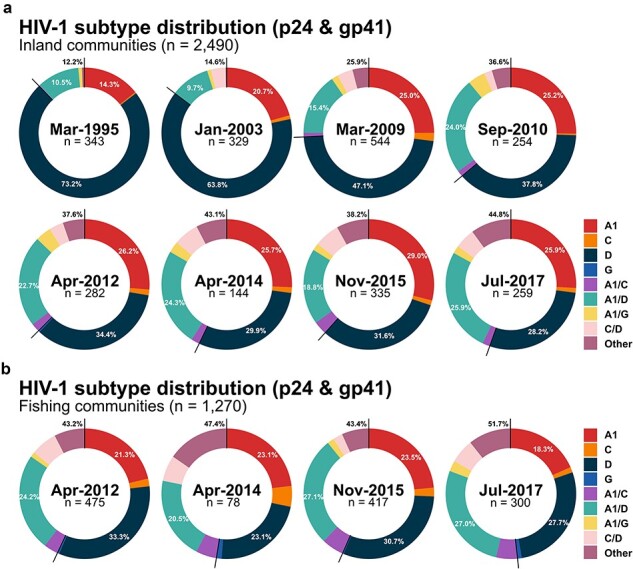
HIV subtype distribution of p24 and gp41 from RCCS participants living with HIV who had available sequence data from both genes in the same survey in (a) 31 inland agrarian and semi-urban trading communities and (b) four hyperendemic Lake Victoria fishing communities by calendar time.

In inland communities, the most common HIV subtypes were A1 and D subtypes, followed by A1/D recombinant viruses, although the proportions of these subtypes varied over calendar time (Fig. 2a; Supplementary Figs S1a,S2a, and S3; and Supplementary Tables S6,S7, and S8). Among participants who had both p24 and gp41 sequence data in the same survey, the proportion of subtype A viruses increased from 14.3% in 1995 to 25.0% until 2009 (*P* < .001), and then leveled off around ∼25% (*P* = .8515). Subtype D viruses consistently declined from 73.2% in 1995 to 28.2% in 2017 (*P* < .001). Similar patterns were observed among participants who had p24 or gp41 sequences available but not necessarily both. Subtype A1 viruses proportionately increased from 16.6% in p24 and 21% in gp41 until 2010 (p24: *P* < .001, gp41: *P* < .001), after which stabilizing around ∼31% in p24 and ∼47% in gp41 between 2012 and 2017 (p24: *P* = .8232, gp41: *P* = .9843). Meanwhile, the proportion of subtype D viruses significantly declined in p24 from 79.6% in 1995 to 44.7% in 2017 (*P* < .001) and in gp41 from 75.9% in 1995 to 38.2% in 2017 (*P* < .001).

Considering inland community participants with subtype data in both genes, the proportion of recombinant viruses increased from 12.2% in 1995 to 44.8% in 2017 (*P* < .001) (Fig. 2a; Supplementary Fig. S3; and Supplementary Table S6). The proportion of A1/D recombinant viruses substantially increased during the earlier surveys from 10.5% in 1995 to 24.0% in 2010 (*P* < .001), after which they leveled off to around 23% (*P* = .2046). Recombinant viruses of more than three different subtypes (e.g. A1/C/D and A/D/G) or rare recombinant forms in our sequence data (e.g. D/G and C/G) also significantly increased over calendar time, from 0.5% in 1995 to 10.0% in 2017 (*P* < .001). By 2017, 44.8% of participants with sequence data in both genes were classified as having an inter-subtype recombinant infection. p24 sequences were significantly more likely to be classified as recombinants over the analysis period. The proportion of recombinants in p24 significantly increased from 2.7% in 1995 to 21.4% in 2017 (*P* < .001) (Supplementary Fig. S1a and Supplementary Table S7) and in gp41 from 2.4% in 1995 to 9.5% in 2017 (*P* < .001) (Supplementary Fig. S2a and Supplementary Table S8). In both genes, the most common recombinant infection was A1/D recombinants.

In Lake Victoria fishing communities, the proportion of viruses classified as either subtype A1 or D did not substantially change between 2012 and 2017 (Fig. 2b; Supplementary Figs S1b, S2b, and S3; and Supplementary Tables S9, S10, and S11). Overall, recombinant viruses were slightly more common in fishing communities than inland communities, but these differences were not significant in either p24 or gp41. Among individuals with HIV sequence data in both genes, the proportions of subtypes A1, D, and A1/D recombinant viruses were generally similar to those in inland communities over the analysis period. Similar to inland communities, recombinants were more frequently detected in p24 than in gp41, and were usually A1/D, A1/C, or C/D recombinants.

We further stratified HIV subtypes by participant demographic and sexual behavior characteristics across three calendar epochs for inland and fishing communities: pre-ART (1994–2004), ART roll-out and expansion (2008–13), and Universal Test and Treatment era (2013–18) (Supplementary Figs S4–S9). Regardless of population subgroups, the proportion of subtype A1 and recombinant viruses increased while subtype D viruses proportionally decreased over calendar time.

### Inter-subtype diversity as measured by SDI

**Figure 3.**
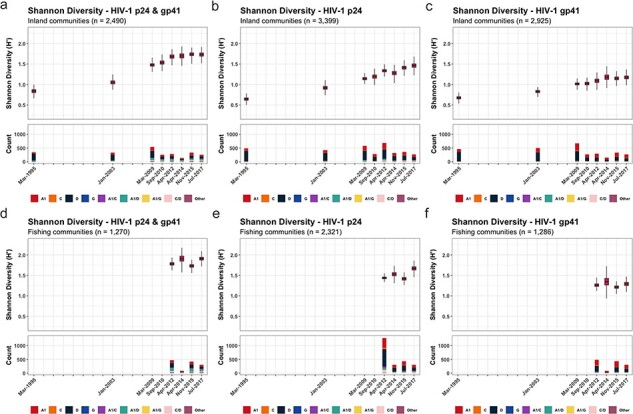
The SDI of p24, gp41, and both p24 and gp41 in 31 inland agrarian and semi-urban trading communities and four Lake Victoria fishing communities between 1995 and 2017.

Next, we evaluated trends in inter-subtype HIV diversity by calculating the SDI in p24 and gp41 genes over calendar time for both inland and fishing communities (Fig. 3). Overall, the SDI increased in p24 through the study period in both inland ($\beta $ = 0.104; 95% CI = 0.092 to 0.116) and fishing communities ($\beta $ = 0.06; 95% CI = 0.028 to 0.091). The SDI also increased in gp41, although no significant changes from 2014 onward were identified in either inland ($\beta $ = −0.006; 95% CI = −0.079 to 0.074) or fishing communities ($\beta $ = −0.037; 95% CI = −0.133 to 0.076). SDI trends were generally similar irrespective of age, sex, occupation, number of sexual partners in the past year, extra-community partners in the past year, or among incident/nonincident cases (Supplementary Figs S10–S16). Notable exceptions included adolescents and young adults (15−24 years) and persons with a recent migration history in inland communities, with these groups exhibiting slightly higher levels of p24 inter-subtype HIV diversity than the rest of the population in the most recent survey period (Supplementary Figs S10 and S14).

### Trends in nucleotide diversity and genetic divergence

**Figure 4.**
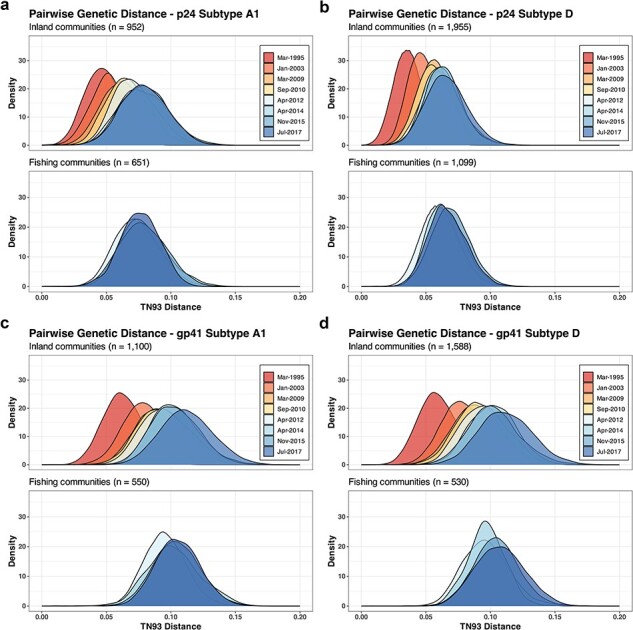
Pairwise TN93 genetic distances of p24 and gp41 subtypes A1 and D in 31 inland agrarian and semi-urban trading communities and four hyperendemic Lake Victoria fishing communities between 1995 and 2017.

Within inland communities, overall nucleotide diversity, irrespective of viral subtype, increased in both genes over the analysis period (Supplementary Fig. S17). Intra-subtype diversity in A1 and D p24 subtypes leveled off after 2014 (Subtype A1: $\beta $ = 0.0027; 95% CI = −0.0007 to 0.0058; Subtype D: $\beta $ = 0.0025; 95% CI = −0.0003 to 0.0043) (Fig. 4). In contrast, A1 and D gp41 nucleotide diversity increased continuously. In fishing communities, p24 nucleotide diversity in subtype A1 increased through 2014 and then decreased, though not significantly ($\beta $ = −0.001; 95% CI = −0.0054 to 0.003), while p24 nucleotide diversity of subtype D increased through 2015 and then decreased ($\beta $ = −0.0063; 95% CI = −0.012 to −0.0005) (Fig. 4 and Supplementary Fig. S18). Similar to inland communities, overall and intra-subtype-specific nucleotide gp41 diversity increased throughout the analysis periods. Nucleotide diversity trends were generally similar across population subgroups (Supplementary Figs S19–S31), although slightly higher overall p24 nucleotide diversity, but not intra-subtype diversity, was observed among younger individuals 15–24 years in inland communities (Supplementary Fig. S19).

We further evaluated population-level HIV genetic divergence by using root-to-tip divergence analyses (Supplementary Fig. S32) and pairwise TN93 distances between subtype reference consensus sequences (A1, C, and D) and HIV sequences in Rakai (Supplementary Fig. S33). Similar to nucleotide diversity, p24 diverged from HIV subtype consensus sequences through 2014 and then leveled off, while gp41 diverged over the analysis period. In the analyses of root-to-tip divergence, higher degrees of genetic variability were observed in gp41 compared to p24 in the same surveys.

Sensitivity analyses were performed to assess the impact of the small sampling fraction of PLHIV on intra-subtype genetic diversity. The medians of nucleotide diversity from 1000 random sequence data sets were similar to the estimates from the full sequence sets in both inland and fishing communities (Supplementary Figs S34 and S35). This similarity suggests that the influence of smaller sampling fractions in later surveys may be minimal. Further, we evaluated the impact of different HIV sequencing protocols on intra-subtype HIV genetic diversity using root-to-tip divergence and linear regression analyses (Supplementary Fig. S36). Although gp41 subtype A1 sequences generated using the Gall protocol showed some deviation, regression lines for most protocols generally overlapped with overall root-to-tip divergence trends, suggesting a limited influence of HIV sequencing protocols on intra-subtype HIV genetic diversity measurement.

### Demographic history of HIV

The posterior estimates of effective population size through time in p24 and gp41 subtypes A1 and D generally showed similar patterns, characterized by a low effective population size during the initial phase of the HIV epidemic, followed by explosive growth until the early 1990s (Supplementary Fig. S37). Notably, trends in the effective population size post-1994 were generally similar to the patterns in nucleotide genetic diversity. While the effective population size in p24 subtype A1 stabilized after 2010, a slight increase was observed in p24 subtype D. However, effective population size in gp41 subtypes A1 and D steadily increased after 1994, consistent with patterns in intra-subtype HIV genetic diversity.

### HIV selection pressure measures

Overall, Tajima’s *D* values were less than 0 in p24 and gp41 over the analysis period, indicating purifying selection in both genes; however, this selection was stronger in p24 than in gp41 (Supplementary Figs. S38 and S39). Similarly, $\omega $ values were less than 1, also indicating a gene-wide purifying selection acting on both genes in subtypes A1 and D viruses (Supplementary Table S12). As in Tajima’s *D* analyses, $\omega $ values in p24 were also lower than those in gp41. In p24, subtype A1 viruses had slightly higher $\omega $ than subtype D viruses, suggesting relatively weaker purifying selection pressure acting on subtype A1. In contrast, $\omega $ values were slightly higher among gp41 subtype D than among gp41 subtype A1, indicating relatively stronger purifying selection acting on subtype A1 gp41.

## Discussion

In this study, we examined longitudinal trends in population-level HIV diversity within 31 inland agrarian and semi-urban trading communities and four hyperendemic Lake Victoria fishing communities in Rakai, Uganda, over a 24-year period, including the introduction and the expansion of HIV prevention and treatment programs. We found that population HIV genetic diversity and divergence increased over calendar time. Increases in HIV genetic diversity were primarily driven by an increasing proportion of inter-subtype recombinant forms and rising intra-subtype gp41 genetic diversity. While Lake Victoria fishing communities are recognized as key populations with a high HIV burden and population mobility (Chang et al. 2016, Kagaayi et al. 2019), neither inter- nor intra-subtype genetic diversity was significantly higher compared to inland communities.

Overall, our HIV genomic sequence data showed that trends in HIV genetic diversity were generally consistent with those from previous analyses conducted in Uganda and eastern Africa. Notably, the proportions of inter-subtype recombinant viruses and their increasing trends, and a decline in subtype D viruses, were remarkably similar to those in previous systematic reviews and molecular studies of HIV sequence data from Uganda (Grant et al. 2020, Hemelaar et al. 2020) and within Africa more broadly (Lee et al. 2017, Hemelaar et al. 2019). Here, we further show that changes in HIV subtype distribution persisted following the extensive scale-up of HIV programs; subtypes A1 and D remained predominant, while the proportion of inter-subtype recombinants significantly increased. While a previous study from the RCCS reported a decline in HIV intra-subtype genetic diversity shortly following ART rollout (Lamers et al. 2020), our findings indicate a consistent increase in genetic diversity in gp41 with stabilizing diversity in p24. The differences in trends in intra-subtype genetic diversity between the previous and current study from the same population, particularly in gp41, may be attributed to the increased sample size in this study (Lamers et al. 2020). The previous study analyzed HIV sequence data from 1364 participants over four surveys, while our study evaluated HIV genetic diversity using data from 3213 individuals in the same four surveys. Additionally, the different HIV subtype assignment methods used in each study may have also affected the observed trends in genetic diversity. Specifically, the RIP with phylogenetic confirmation is more robust for HIV subtype assignment than the HIV subtyping tools used in the previous study, potentially resulting in differences in HIV subtype assignment and intra-subtype genetic diversity trends.

Between 1994 and 2018, we observed a substantial decrease in the proportion of subtype D viruses and an increasing proportion of subtype A1 viruses among individuals living in inland communities. These trends are consistent with findings in previous studies from Uganda (Conroy et al. 2010, Hemelaar et al. 2019, Lamers et al. 2020) and are likely attributed to pathogenic and clinical characteristics of subtype A1 and D viruses. For example, infection with HIV subtype D is associated with a rapid decrease in CD4+ cell count compared to subtype A1 and other non-D subtypes, resulting in faster progression to AIDS and earlier initiation of ART. Thus, there is potentially a shorter amount of time for subtype D viruses to be transmitted than subtype A1 or other subtypes (Kaleebu et al. 2002, Baeten et al. 2007, Kiwanuka et al. 2008, Ssemwanga et al. 2013). Additionally, in a previous study in Uganda, individuals in HIV serodiscordant couples whose partners were infected with subtype D viruses were found to be at a lower risk of acquiring HIV compared to those with subtype A1 (Kiwanuka et al. 2009). The higher rate of HIV disease progression and lower transmissibility of subtype D compared to subtype A1 or other subtypes may have led to the observed patterns in the proportion of subtype A1 and D viruses in this study.

We further showed that increases in HIV genetic diversity were largely driven by an increase in HIV inter-subtype recombinant infections. In both inland and fishing communities, the most common recombinants were A1/D recombinants, consistent with previous molecular and surveillance studies in Uganda (Eshleman et al. 2002, Harris et al. 2002, Yirrell et al. 2002, Arroyo et al. 2006, Conroy et al. 2010, Capoferri et al. 2020, Grant et al. 2020, Lamers et al. 2020). Interestingly, despite the low prevalence of pure subtype C viruses in our HIV sequence data, we observed a substantial increase in inter-subtype C recombinants (A1/C and C/D) in p24. This might be attributed to introduction of subtype C, which accounted for 15% of HIV infections in eastern Africa by 2015 (Hemelaar et al. 2019). A previous study in Lake Victoria fishing communities in southern Uganda indicated a higher HIV prevalence among in-migrating populations from other geographic locations, with the highest rates observed among migrants from Tanzania (Grabowski et al. 2020). Another molecular study, conducted prior to ART rollout in Rakai, revealed that 19% of inter-subtype recombinants were A1/C and C/D viruses in trading centers along Masaka road connecting Kampala to the border with Tanzania (Arroyo et al. 2006). These studies suggest that there might be a continuous influx of subtype C viruses or C recombinants into Uganda (Bbosa et al. 2019, Grabowski et al. 2020), resulting in inter-subtype viral mixing and increasing HIV genetic diversity.

Increasing HIV subtype diversity might be partly driven by individuals acquiring multiple infections of different HIV subtypes. Previous analyses from the same population revealed that individuals who remain viremic tended to have substantially higher levels of HIV-related risk behaviors compared to those who were virally suppressed (Brophy et al. 2021, Kennedy et al. 2023), potentially increasing the risk of coinfections and superinfections. However, our analyses of inter-subtype genetic diversity revealed no significant differences in levels of recombinant infection across various population subgroups, including those with and without a recent history of migration or those having extra-community sexual partners. In particular, despite high levels of HIV-related risk behaviors and population mobility in fishing communities, inter-subtype diversity was not significantly higher compared to inland communities. This might be due to widespread viral population mixing and pervasive inter-subtype recombination in the generalized and diverse HIV epidemic in Uganda (Lee et al. 2017, Grant et al. 2020). In analyses of HIV subtype distribution from individuals with HIV sequence data in both genes, the proportion of recombinant viruses in 2017 was 44.8% in inland communities and 51.7% in fishing communities. These estimates are consistent with findings in a previous study of near full-length genomes of HIV in Uganda, indicating that nearly 50% of viruses were unique recombinant forms (Grant et al. 2020). Inter-subtype recombination via coinfections and superinfections is common in Rakai (Taylor and Korber 2005, Redd et al. 2012, 2013) and potentially driving HIV diversity in this region.

Our data showed increasing trends in intra-subtype diversity, the effective population size, and genetic divergence in gp41 irrespective of population subgroups. We also found higher Tajima’s *D* and $\omega $ values in gp41, consistent with the previous analyses in Rakai and elsewhere (Alizon and Fraser 2013, Raghwani et al. 2018). These findings suggest that diversifying selection is acting more intensively on gp41 (or *env*), possibly due to its critical role in immune evasion, while p24 (or *gag*) is likely under stronger functional constraints (Lin et al. 2019). However, it is unclear why subtype-specific genetic diversity in p24 leveled off following the widespread expansion of HIV treatment and prevention programs. One possibility is that the success of HIV intervention programs might limit the opportunities for mutation and recombination events. Continued molecular surveillance with full-length HIV genome sequence data from diverse geographic locations may be needed to understand the changing dynamics of HIV and the long-term impact of these HIV programs.

Increasing genetic diversity in gp41 may have significant implications for vaccine development. Various candidate HIV vaccines have either failed or demonstrated limited efficacy partly due to high levels of HIV genetic diversity, which has led to calls for developing new vaccines that elicit bNAb responses (Burton and Hangartner 2016, Sadanand et al. 2016, Burton 2019, Steichen et al. 2019). bNAbs are capable of recognizing and neutralizing a breadth of HIV subtypes by binding to relatively conserved regions on the *env* protein. The primary challenge in developing vaccines is to reliably induce these bNAbs responses, which could be complicated by the high mutation rate and variability on the *env* gene. For example, 2F5 and 4E10 are two extensively studied bNAbs that target the membrane-proximal external region (MPER) of the *env* protein, a part of the region in our gp41 sequence analyses (Ofek et al. 2004, Cardoso et al. 2005, Brunel et al. 2006, Alam et al. 2007). Increasing genetic diversity in gp41, especially around the MPER or other potential target regions in the whole *env* protein, may pose significant challenges for vaccine design, as genetic changes through mutations or inter-subtype recombination in these regions may allow the viruses to escape bNAb responses. Our findings highlight the need for a multifaceted approach in HIV vaccine development that not only focuses on the robust induction of bNAbs but also considers the rapid genetic shifts in gp41 though enhanced molecular surveillance and monitoring.

There are several limitations to this study. First, we used only two short genomic regions of HIV for our analyses; thus, it is possible that our assessment of HIV subtype distribution might be biased, and the extent of HIV genetic diversity is likely underestimated. Previous analyses of near full-length genomes from Uganda estimated that 30%–50% of viruses were inter-subtype recombinants (Harris et al. 2002, Lee et al. 2017, Grant et al. 2020). In particular, the frequency of recombination breakpoints tends to be lower in the essential regions (e.g. *gag, pol*, and *env*), with the *env* gene exhibiting the lowest frequency (Lee et al. 2017, Grant et al. 2020). This could explain the relatively lower proportion of inter-subtype recombinant viruses in gp41 compared to p24 in our data. Additionally, intra-subtype recombination, which may occur just as frequently as inter-subtype recombination, could significantly contribute to genetic variation within subtypes, but we did not evaluate it here. Third, we only analyzed HIV consensus sequence data, and the recombinant sequences identified in this study may potentially result from dual infections. Further analyses at the read level may be needed to identify the extent of recombination events. Fourth, we did not formally evaluate drivers of genetic diversity. Increasing intra-subtype HIV genetic diversity might be partly due to viral migration from regions outside RCCS communities and also other factors including intra-subtype recombination, within-host evolution, lifelong infection, and the incomplete uptake of suppressive therapy. Mathematical models that account for these various factors may be necessary for a comprehensive understanding of the underlying causes of increasing HIV genetic diversity in future studies. Fifth, we utilized HIV sequence data generated from different sequencing methods and protocols. These changes in sequencing methods may have influenced our assessment of intra-subtype HIV genetic diversity. However, our root-to-tip divergence and regression analyses suggested a limited impact of different sequencing protocols on observed trends. Sixth, our results may have been impacted by sampling biases. Young individuals and men in Rakai generally had lower participation rates in the RCCS (Grabowski et al. 2020). However, these population subgroups were more likely to be sequenced, particularly in the later survey rounds, and thus these individuals living with HIV were over-represented in our sequence data. Furthermore, HIV sequence data in the more recent surveys with higher ART coverage may not represent overall population viral diversity, as only individuals with viral loads greater than 1000 copies at the time of sampling or not self-reporting ART use were sequenced. Lastly, we used self-reported data to evaluate the HIV genetic diversity among individuals who had HIV-related high-risk behaviors, which may be influenced by reporting bias.

In conclusion, we observed continued HIV evolution, with a growing number of recombinant viruses and increasing intra-subtype evolution of gp41. Overall, HIV genetic diversity increased in both inland agrarian and semi-urban trading communities and hyperendemic Lake Victoria fishing communities, irrespective of population subgroups. Comprehensive and wide-ranging molecular surveillance is essential for a better understanding of HIV dynamics driving population HIV evolution and the development of new HIV vaccines and treatments for epidemic control.

## Supplementary Material

veae065_Supp

## Data Availability

The data underlying this article are available in the GenBank Nucleotide Database at https://www.ncbi.nlm.nih.gov/genbank/, and can be accessed with the GenBank accession numbers PQ289642-PQ295210 and listed in Supplementary table S13.
